# Supplementary material for: Harnessing landrace diversity empowers wheat breeding
Source: Nature. 2024 Jun 17;632(8026):823–31. doi: 10.1038/s41586-024-07682-9 (PMC11338829; doi:10.1038/s41586-024-07682-9)
Supplement: Supplementary file 2 — Reporting Summary [file 41586_2024_7682_MOESM2_ESM.pdf]

nature portfolio

Corresponding author(s): Shifeng Cheng
Simon Griffths

Last updated by author(s): Sep 16, 2023

Reporting Summary

Nature Portfolio wishes to improve the reproducibility of the work that we publish. This form provides structure for consistency and transparency in reporting. For further information on Nature Portfolio policies, see our Editorial Policies and the Editorial Policy Checklist.

Statistics

For all statistical analyses, confirm that the following items are present in the figure legend, table legend, main text, or Methods section.

n/a	Confirmed	
☐	☒	The exact sample size (*n*) for each experimental group/condition, given as a discrete number and unit of measurement
☐	☒	A statement on whether measurements were taken from distinct samples or whether the same sample was measured repeatedly
☐	☒	The statistical test(s) used AND whether they are one- or two-sided<br>*Only common tests should be described solely by name; describe more complex techniques in the Methods section.*
☒	☐	A description of all covariates tested
☐	☒	A description of any assumptions or corrections, such as tests of normality and adjustment for multiple comparisons
☐	☒	A full description of the statistical parameters including central tendency (e.g. means) or other basic estimates (e.g. regression coefficient) AND variation (e.g. standard deviation) or associated estimates of uncertainty (e.g. confidence intervals)
☐	☒	For null hypothesis testing, the test statistic (e.g. *F*, *t*, *r*) with confidence intervals, effect sizes, degrees of freedom and *P* value noted<br>*Give P values as exact values whenever suitable.*
☒	☐	For Bayesian analysis, information on the choice of priors and Markov chain Monte Carlo settings
☒	☐	For hierarchical and complex designs, identification of the appropriate level for tests and full reporting of outcomes
☐	☒	Estimates of effect sizes (e.g. Cohen's *d*, Pearson's *r*), indicating how they were calculated

Our web collection on statistics for biologists contains articles on many of the points above.

Software and code

Policy information about availability of computer code

Data collection

No software used for data collection.

Data analysis

Alignment and SNP calling:
Alignment: BWA-MEM (v0.7.17)
Mark duplicate: picard (v 2.20.3-SNAPSHOT)
Sort Alignment file: SAMtools (v1.9)
SNP/Indel Calling: GATK (v4.1.2)
SNP/Indel filtering: (https://github.com/ShifengCHENG-Laboratory/WWWG2B/tree/main/00.SNP_calling_and_QC)
LD pruning: PLINK (v1.90b6.7)

Population structure analysis:
Phylogenetic tree: iqtree (v1.6.9); rapidnj(Version 2.3.2)
Tree visualisation: iTOL (https://itol.embl.de/)
Structure: ADMIXTURE (v1.3.0)
Genetic diversity (π): vcftools (v0.1.13)
Population differentiation (FST): vcftools (v0.1.13)
identity-by-state matrix: PLINK (v1.90b6.7)
PCA: Python-sklearn (v0.24.2)
t-SNE: Python-sklearn (v0.24.2)

Wheat HapMaP construction:

Block identification: PLINK (v1.90b6.7)
Block connection: (https://github.com/ShifengCHENG-Laboratory/WWWG2B/tree/main/01.HAPMAP_pipeline)
Haplotype assignment: HAPPE (v0.1.4), https://github.com/fengcong3/HAPPE

GWAS study:
Calculate kinship and perform GWAS: GEMMA (v0.98.1)
Manhattan plot and QQ plot: (https://github.com/ShifengCHENG-Laboratory/WWWG2B/tree/main/04.GWAS_pipeline)
Local details and LD: LDBlockShow (v1.40)

NAM imputation:
Imputation: (https://github.com/ShifengCHENG-Laboratory/WWWG2B/tree/main/03.NAM_imputation_pipeline)
Merge VCF: bcftools (v1.9)

CNV identification:
Blastn (v 2.8.1+)
In-house pipeline(https://github.com/ShifengCHENG-Laboratory/WWWG2B/tree/main/07.CNV_pipeline)

k-mer databases count from raw reads and genome assemblies:
kmerGWAS pipeline https://github.com/voichek/kmersGWAS
Jellyfish v.2.2.6
KMC v3.0.1

IBSpy variations and Long Range haplotypes:
The implementation of the complete pipeline is in the final version of IBSpy (v.0.4.6) in https://github.com/Uauy-Lab/IBSpy
Affinity Propagation (AP) methods and the API: https://scikit-learn.org/stable/modules/generated/sklearn.cluster.AffinityPropagation.html
AP clusters were scored by Silhouette Coefficient (SC) score and the API: https://scikit-learn.org/stable/modules/generated/sklearn.metrics.silhouette_score.htm
Syntenic windows among wheat genome references were developed in (Brinton et al. Communications Biology, 3, 712)
Minimum landrace path: https://github.com/Uauy-Lab/MLP_finding.

Sequencing data quality control:
Comparison of 35k Axiom array with sequence data: https://github.com/JIC-CSB/WatSeqAnalysis/tree/master/qc_vs_iselect

Statistical analyses
R software suite (version 4.2)
Pearson correlation coefficient (r) was calculated using R package corr v0.4.4 and corrplot v0.92.
ANOVA has been calculated as a linear model with the genotype as a factor, using base function lm.
AMMI was calculated using base functions aov and svd following the code from https://journal.r-project.org/articles/RN-2007-003/RN-2007-003.pdf

Genetic map construction:
Mapdisto (version 1.7) for KASP genotypes and R package ASMap (version 1.6) for 35k Wheat Breeders' array genotyping.

QTL analysis
R software suite (v3.6.1) package qtl (v1.5)

Triticum aestivum Next Generation array
Scripts for the design of the TaNG genotyping array: https://github.com/pr0kary0te/GenomeWideSNP-development

Standardization of phenotypic data:
Crop Ontology Curation tool (https://cropontology.org/)

Geographic distance:
R package geosphere (v1.5-18)

For manuscripts utilizing custom algorithms or software that are central to the research but not yet described in published literature, software must be made available to editors and reviewers. We strongly encourage code deposition in a community repository (e.g. GitHub). See the Nature Portfolio guidelines for submitting code & software for further information.

Data

Policy information about availability of data

All manuscripts must include a data availability statement. This statement should provide the following information, where applicable:

- Accession codes, unique identifiers, or web links for publicly available datasets
- A description of any restrictions on data availability
- For clinical datasets or third party data, please ensure that the statement adheres to our policy

All whole-genome sequence data has been deposited at the National Genomics Data Center (NGDC) Genome Sequence Archive (GSA) (https://ngdc.cncb.ac.cn/gsa/search?searchTerm=CRA012590), with BioProject accession number PRJCA019636, and with GSA Accession ID: CRA012590. Variation matrix and annotations, wheat HapMap, phenotyping data, genetic maps with genotype scores, association genetics analyses, the developed tagSNPs and KASP markers were deposited in WWWG2B breeding portal (https://wwwg2b.com). IBSpy variations tables, haplotypes, long-range tilling paths, variant files (VCF) and all raw phenotypic data are available online (https://wwwg2b.com/dataAvailable, https://wwwg2b.com/toolIndex/academic and https://opendata.earlham.ac.uk/wheat/under_license/toronto/WatSeq_2023-09-15_landrace_modern_Variation_Data/). Publicly available sequencing data were obtained from SRA accessions SRP114784, PRJNA544491, PRJEB37938, PRJNA492239, PRJNA528431, PRJEB39558, PRJEB35709 and from the NGDC database project CRA005878.

The IWGSC RefSeq is download from https://urgi.versailles.inra.fr/download/iwgsc/IWGSC_RefSeq_Assemblies/v1.0/ .

Variation matrix (VCF files) and annotations, wheat haplotype map, phenotyping data, association genetics analyses including the identified QTL and MTA, the developed tagSNPs and KASP markers were deposited in WWWG2B breeding portal (https://wwwg2b.com/).

Variation matrix (VCF files) for the 1051 accessions are also available at:
https://wwwg2b.com/dataAvailable/SNP_matrix/ and https://opendata.earlham.ac.uk/wheat/under_license/toronto/
WatSeq_2023-09-15_landrace_modern_Variation_Data/WatSeq_VCF_ChineseSpringRefSeqv1.0/

IBSpy variations tables of the 1051 accessions used in this study are available at:
https://wwwg2b.com/dataAvailable/IBSpy_variations_10WheatGenomes/ and https://opendata.earlham.ac.uk/wheat/under_license/toronto/
WatSeq_2023-09-15_landrace_modern_Variation_Data/IBSpy_variations_10WheatGenomes/

IBSpy-based haplotypes based on the Chinese Spring reference sequence are available at:
https://wwwg2b.com/dataAvailable/IBSpy_haplotypes_ChineseSpring/ and https://opendata.earlham.ac.uk/wheat/under_license/toronto/
WatSeq_2023-09-15_landrace_modern_Variation_Data/IBSpy_haplotypes_ChineseSpring/

Syntenic blocks between 10+ Wheat genome references used for Affinity Propagation haplotype calls are available at:
https://wwwg2b.com/dataAvailable/Syntenic_blocks_by_chromosome/ and https://opendata.earlham.ac.uk/wheat/under_license/toronto/
WatSeq_2023-09-15_landrace_modern_Variation_Data/Syntenic_blocks_by_chromosome/

Raw phenotypic data is available at https://wwwg2b.com/dataAvailable/Watseq_phenotype_data/, https://grassroots.tools/fieldtrial/ and https://opendata.earlham.ac.uk/wheat/under_license/toronto/WatSeq_2023-09-15_landrace_modern_Variation_Data/WatSeq_phenotypic_data/ (field and glasshouse data). Yellow rust resistance scores for Watkins natural populations in Ethiopia and Kenya field trials are available here: https://doi.org/10.5281/zenodo.8349020.

35k Wheat Breeder's array genotype data of RILs and NILs is available at: https://www.cerealsdb.uk.net/cerealgenomics/CerealsDB/array_info.php

Additional datasets are provided in the Supplementary Tables.

Research involving human participants, their data, or biological material

Policy information about studies with human participants or human data. See also policy information about sex, gender (identity/presentation), and sexual orientation and race, ethnicity and racism.

Reporting on sex and gender	not applicable
Reporting on race, ethnicity, or other socially relevant groupings	not applicable
Population characteristics	not applicable
Recruitment	not applicable
Ethics oversight	not applicable

Note that full information on the approval of the study protocol must also be provided in the manuscript.

Field-specific reporting

Please select the one below that is the best fit for your research. If you are not sure, read the appropriate sections before making your selection.

☒ Life sciences ☐ Behavioural & social sciences ☐ Ecological, evolutionary & environmental sciences

For a reference copy of the document with all sections, see nature.com/documents/nr-reporting-summary-flat.pdf

Life sciences study design

All studies must disclose on these points even when the disclosure is negative.

Sample size	The Watkins collection consists of 827 individuals. Unreplicated field trial data was used for GWAS. The diversity subset of the Watkins collection, grown in Egypt, contained 300 Watkins lines and was used for GWAS. The RIL population size was near 94 RILs for most of the 73 populations and was used for QTL mapping. In a very few extreme cases, QTL mapping was conducted with as low as 88 RILs. We assume that a small population size will lead to a higher false positive rate in QTL mapping. We counteract this problem in our research strategy by either a repetition of the QTL experiment in another season or by checking QTL effects in NIL validation trials. Furthermore, we use several populations grown at the same site and the same season, for NAM-GWAS analysis. Sample size in the NAM GWAS was between several hundred to several thousand RILs.
Data exclusions	The genotyping of three RIL populations (ParW444, ParW264, ParW313) out of 112 populations, revealed unexpected genotypes, which could only be explained by a mistake in the crossing programme. The populations and collected data were discarded, resulting in 109 RIL populations in the study.

NIL families Fam-013 to Fam-016 and Fam-023 lost all members with a second allele due to a drilling error in the multiplication trial. These families were removed from the data analysis.

Data from three NIL lines of family Fam-008 was excluded from the analysis because they showed a striking dwarf growth habit in the field that was very different to the rest of the germplasm.

Data from two lines WL0019 and WL0026 from the SB_H18 TK trial was excluded from the analysis of the Q7B-PH grain yield effect, as the plant height effect for all three replicates suggested that the samples had been swapped and it was not possible to go back to the original records.

Replication

Field trials with the 827 Watkins accessions were grown in a randomised, unreplicated augmented block design with 10% 'check' varieties. Similarly, glasshouse trials of the 827 Watkins acessions, to confirm specific effects like yellow rust resistance or glume colour, were conducted in randomised but unreplicated trials, due to the size of the collection. Two Watkins trials in China were grown in a randomised split-plot design with two Nitrogen fertiliser treatments and two replicates each. Initial seed multiplication exercises of the 127 RIL populations, each with 88-102 individuals, were unreplicated and were grown in augmented block design with 20% check varieties. TK trials at JIC, consisting of NIL families of two to 8 NILs with two contrasting alleles, were grown in augmented block design with 10% check varieties in the multiplication trial. After multiplication, TK trials were grown in three replicates and in randomised block design at JIC, RH and SB. All RIL trials at RH and SB, were grown in randomised block design with three replicates. Specific NIL trials, comparing an individual NIL pair, were grown in randomised block design with three replicates, or even five replicates for the RHT8 trials in Spain and Serbia. Yellow rust trials of RIL populations (90-94 RILs each) at commercial breeding stations were grown in randomised unreplicated trials, with two time points of disease scoring.

Randomization

All field trial design included randomisation of the genotypes.

Blinding

Phenotypic measurements on the Watkins, RILs, and NILs were taken independently by different scientists, across locations, and without knowledge of the underlying identifier of each accession. Hence all phenotypic measurements were blinded.

Reporting for specific materials, systems and methods

We require information from authors about some types of materials, experimental systems and methods used in many studies. Here, indicate whether each material, system or method listed is relevant to your study. If you are not sure if a list item applies to your research, read the appropriate section before selecting a response.

Materials & experimental systems

n/a	Involved in the study
☒	☐ Antibodies
☒	☐ Eukaryotic cell lines
☒	☐ Palaeontology and archaeology
☒	☐ Animals and other organisms
☒	☐ Clinical data
☒	☐ Dual use research of concern
☐	☒ Plants

Methods

n/a	Involved in the study
☐	☒ ChIP-seq
☒	☐ Flow cytometry
☒	☐ MRI-based neuroimaging

Plants

Seed stocks

All the germplasm used in this study is conserved in the UKRI-BBSRC Germplasm Resources Unit (GRU) National Capability at JIC. The seed and passport data is available on https://www.seedstor.ac.uk/.

The progenitor landrace populations (Watkins Historic Collection) are available on https://www.seedstor.ac.uk/search-browseaccessions.php?idCollection=4

The derived 827 sequenced Watkins landraces seed stocks are available on https://www.seedstor.ac.uk/search-browseaccessions.php?idCollection=39

The 208 modern elite wheats used were sourced from various SeedStor collections and can be viewed and ordered collectively as a Compiled Accession List at https://www.seedstor.ac.uk/search-custom.php

Pure stocks of the seed harvested from a single DNA sequenced plant (gold standard stocks) are kept for reference in the GRU and were shared with AGIS. Progeny of these were multiplied in greenhouse with bagged ears for preventing cross pollination and handled following international Genebank Standards (Rome 2014)

Novel plant genotypes

The BBSRC Designing Future Wheat - Recombinant Inbred Lines (RILs) Nested Association Mapping panel (DFW - NAM) comprising 8,359 greenhouse grown cellophane bagged hand threshed seed stocks, deposited in the GRU as part of this work. It includes the

reported 73 RIL populations. Seed stocks are available on https://www.seedstor.ac.uk/search-browseaccessions.php?idCollection=47

The DFW Wheat Academic Toolkit pre breeding germplasm collection of 1,845 lines that were deposited in the GRU as part of this work include all the Near Isogenic Lines (NILs), reported in this study. SeedStock are available on https://www.seedstor.ac.uk/search-browseaccessions.php?idCollection=40

Authentication

High heritability traits (height, heading date, glume colour, seed appearance, presence of awns, length of awns) are confirmed as a standard procedure for all glasshouses grown stocks that were available on SeedStor collections for this study. Seed and ear morphology are compared by the genebank curator to the previously grown seed lot and sampled ear. Plant morphology traits are compared to the previous regeneration data records, dating back to the early 1970s for modern varieties and to the original century old grouping in case of Watkins landrace collection. The purified Watkins landrace accessions and the modern wheat panel were genotyped with Axiom 35K high density genotyping and SNP calls checked against the sequence generated in this study (see Methods). NILs were also subjected to Axiom 35K genotyping. RILs are genotypes with Axiom 35K Breeder array or KASP markers.

To ensure that field plots are composed of the intended lines, they were scored for morphological and high heritability traits such as height, heading, glume colour, and the presence of awns. Awns were particularly useful as Paragon (common NAM and Recurrent backcross parent) carried the B1 awn inhibiting allele on chromosome 5A whereas the majority of Watkins accessions are awned. Trait values were correlated between experiments and QTL mapped (e.g., Awns to B1 on chromosome 5A, height to major QTL on chromosome 6A, glume colour to chromosome 1B). In the event of any doubt, small sets of diagnostic KASP markers were used to genotype ~8 seeds from each field plot. We estimate 2-5% cross contamination of field plots harvested by plot combine harvesters (This was tested and confirmed in 2021 in five breeder and academic institute sites evaluating the DFW stocks by counting off-types in 6-metre plots of F5 NILs). Seed was not multiplied from field plots for more than two generations; new "pure" stocks were taken from glasshouse regenerated plants (bags placed over spike before anthesis to prevent cross pollination, see Methods).

ChIP-seq

Data deposition

☐ Confirm that both raw and final processed data have been deposited in a public database such as GEO.

☐ Confirm that you have deposited or provided access to graph files (e.g. BED files) for the called peaks.

Data access links
May remain private before publication.

For "Initial submission" or "Revised version" documents, provide reviewer access links. For your "Final submission" document, provide a link to the deposited data.

Files in database submission

Provide a list of all files available in the database submission.

Genome browser session
(e.g. UCSC)

Provide a link to an anonymized genome browser session for "Initial submission" and "Revised version" documents only, to enable peer review. Write "no longer applicable" for "Final submission" documents.

Methodology

Replicates

Describe the experimental replicates, specifying number, type and replicate agreement.

Sequencing depth

Describe the sequencing depth for each experiment, providing the total number of reads, uniquely mapped reads, length of reads and whether they were paired- or single-end.

Antibodies

Describe the antibodies used for the ChIP-seq experiments; as applicable, provide supplier name, catalog number, clone name, and lot number.

Peak calling parameters

Specify the command line program and parameters used for read mapping and peak calling, including the ChIP, control and index files used.

Data quality

Describe the methods used to ensure data quality in full detail, including how many peaks are at FDR 5% and above 5-fold enrichment.

Software

Describe the software used to collect and analyze the ChIP-seq data. For custom code that has been deposited into a community repository, provide accession details.
